# Technical Advances to Study Extracellular Vesicles

**DOI:** 10.3389/fmolb.2017.00079

**Published:** 2017-11-28

**Authors:** Paula Carpintero-Fernández, Juan Fafián-Labora, Ana O'Loghlen

**Affiliations:** Epigenetics and Cellular Senescence Group, Blizard Institute, Barts and The London School of Medicine and Dentistry, Queen Mary University of London, London, United Kingdom

**Keywords:** extracellular vesicles (EVs), exosomes, microvesicles (MVs), technique, methods, novel

## Abstract

Extracellular vesicles are a heterogeneous and dynamic group of lipid bilayer membrane nanoparticles that can be classified into three different groups depending on their cellular origin: exosomes, microvesicles, and apoptotic bodies. They are produced by different cell types and can be isolated from almost all body fluids. EVs contain a variety of proteins, lipids, nucleic acids, and metabolites which regulate a number of biological and pathological scenarios both locally and systemically. Different techniques have been described in order to determine EV isolation, release, uptake, and cargo. Although standard techniques such as immunoblotting, fluorescent microscopy, and electron microscopy are still being used to characterize and visualize EVs, in the last years, more fine-tuned techniques are emerging. For example, EV uptake can be specifically determined at a single cell level using the Cre reporter methodology and bioluminescence based-methods reports have been employed to determine both EV release and uptake. In addition, techniques for cargo identification have also enormously evolved during these years. Classical mass spectrometry and next generation sequencing have been used in the past, but nowadays, advances in these tools have facilitated a more in depth characterization of the EV content. In this review, we aim to assess the standard and latest technical advances for studying EV biology in different biological systems.

During the past decades, extracellular vesicles (EVs) have been recognized as potent vehicles of non-cell autonomous intercellular communication in different model systems compared to cell-to-cell communication (Kramer-Albers and Hill, 2016; Tkach and Thery, 2016; O'Loghlen, 2017). The term EVs comprises a highly heterogeneous and dynamic group of lipid bilayer membrane vesicles that can be classified into three main groups: exosomes, microvesicles (MVs), and apoptotic bodies. Exosomes range in size from 30 to 120 nm in diameter and are generated via activation of the endocytic pathway forming multivesicular bodies (MBV), which can later fuse with the plasma membrane and be released to the extracellular environment. Microvesicles (MVs) are larger vesicles with a size between 100 and 1,000 nm and they are formed as the result of the outward budding of the plasma membrane. The third category of EVs are apoptotic bodies that are formed as a result of the induction of cellular apoptosis and comprise a size ranging between 100 and 5,000 nm (Colombo et al., 2013).

It is nowadays well documented that EVs are involved in numerous physiological and pathophysiological processes (Tkach and Thery, 2016). The fact that they are lipid particles involved in signaling during the progression of several diseases forms an attractive basis to use them as potential disease progression biomarkers (Skog et al., 2008; Cocucci and Meldolesi, 2015). Due to that, in the last years different techniques have been developed in order to identify the cellular origin, molecular composition, cargo and uptake of EVs.

In this review, we aim to provide a brief review of the standard and technical advances used to study MVs and exosomes. Thus, we will use the general term EVs to cover both exosomes and MVs in the text.

## Advances in techniques to identify and study EVs

### EV isolation techniques

**Figure 1:**
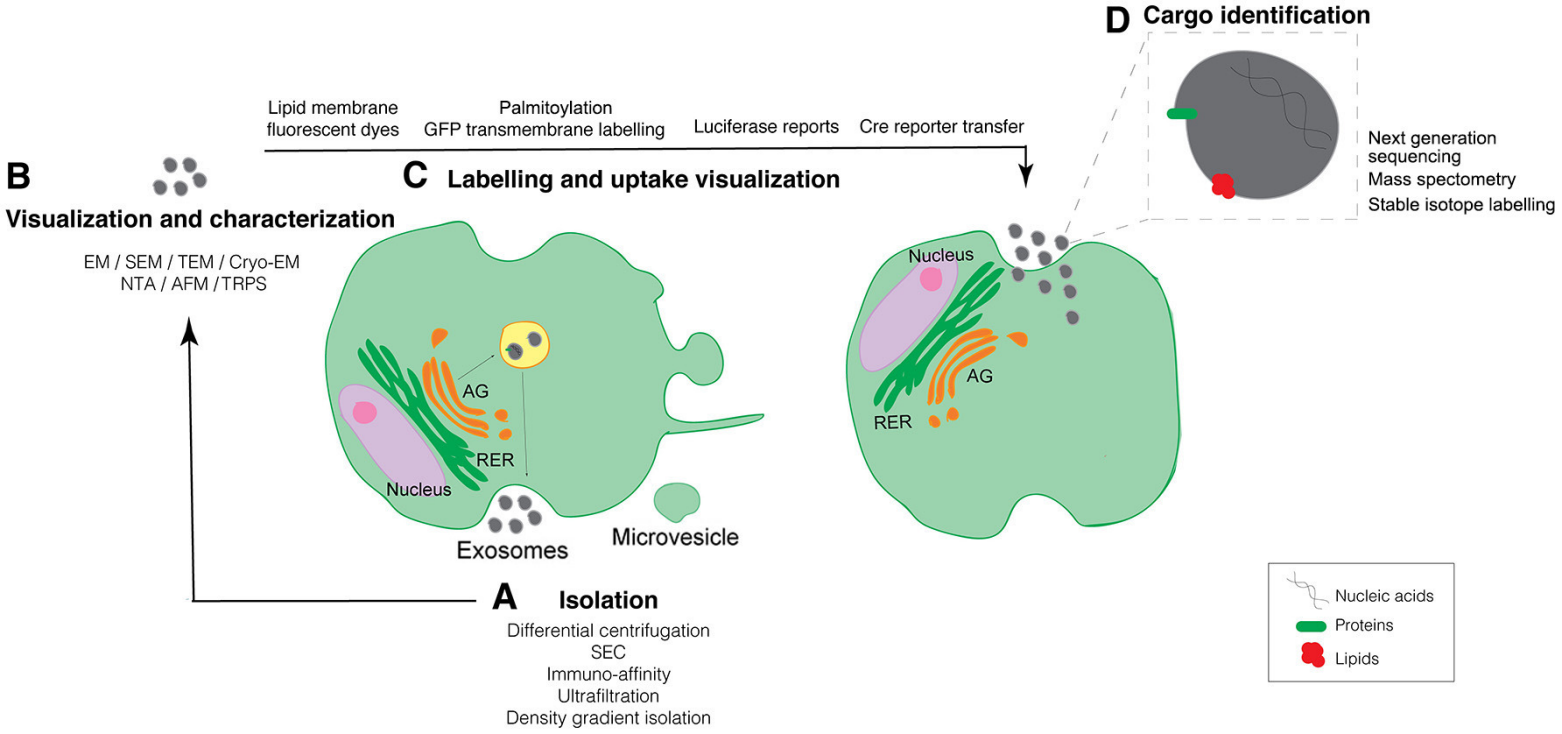
Schematic representation of established and new evolving techniques used for the study of EV biology organized across the biogenesis and uptake of EVs. **(A)** Techniques to isolate EVs. **(B)** Methods to visualize and characterize purified EVs. **(C)** Techniques to allow for the EVs labeling and uptake visualization. **(D)** Identification of EV cargo. GA, Golgi Apparatus; RER, Rough Endoplasmic Reticulum; SEC, Size Exclusion Chromatography; EM, Electron Microscopy; SEM, Scanning Electron Microscopy; TEM, Transmission Electron Microscopy; Cryo-EM, Cryo-Electron Microscopy; NTA, Nanoparticle Tracking Analysis; TRSP, Tunable resistive pulse-sensing; AFM, Atomic Force Microscopy.

Almost all cell types in culture release EVs and, as a matter of fact, EVs can be isolated from many types of body fluids including blood, urine, saliva, and milk (Tkach and Thery, 2016). EVs contain a variety of different biomolecules in their lumen such as proteins, nucleic acids (both RNAs and DNA), metabolites, and lipids that play important roles in cell-to-cell communication (Kramer-Albers and Hill, 2016). Whereas, nowadays many research groups are focused on defining EV composition we have to take into account that the isolation process is one of the most challenging approaches (Mateescu et al., 2017). Differential centrifugation, ultrafiltration, size exclusion chromatography (SEC), immuno-affinity, and density gradient isolation are frequent methods used for EVs isolation (Figure 1A). However, each of these methods have their own limitations ranging from co-isolating contaminants—comprising non-vesicular proteins, lipids, and nucleic acids—to low EV recovery. Nowadays, most groups use a combination of some of the above isolation techniques to overcome the pitfalls of individual isolation techniques (Gardiner et al., 2016). An additional setback of the previously described isolation methods is the lack of a specific tool to isolate and determine particular EV subpopulations. It's been recently shown that the functionality of EVs can highly vary depending on their heterogeneity (Tkach et al., 2017), therefore novel techniques that allow the user to isolate particular EV subpopulations would be extremely advantageous.

To date, there is no ideal single isolation technique and the development of novel methodologies to increase EV recovery and purity, including the possibility to identify individual particular subpopulations will highly benefit not only the scientific community but also the clinical application of EVs as disease biomarkers.

### Techniques to determine EV visualization and characterization

EVs are released from cells by diverse mechanisms depending on their mode of cellular biogenesis and can be taken up by almost all cells (Colombo et al., 2014). It is known that EVs can either bind to the plasma membrane, activating specific signaling pathways or enter into recipient cells by either membrane fusion or by different mechanisms of endocytosis (Thery et al., 2009). The merging of the EV's cargo with the cellular cytoplasmic compartment influences the behavior of the cells taking up the EVs. Therefore, it is important to determine cargo uptake by the recipient cell in order to associate EV uptake with functionality.

The most frequently used techniques to characterize EVs are immunoblotting and antibody specific enzyme-linked immunosorbent assay (ELISA). However, none of these methods give any information on the EV structure, concentration or heterogeneity. Furthermore, immuno-affinity beads capture and detection by fluorescence-activated cell sorting (FACS) allows the detection of individual EV subpopulations but does not give an overall view of the EV heterogeneous population (Ostrowski et al., 2010).

In order to advance our understanding of EV biology, accurate methods that allow us to quantify and visualize single EV particles are needed. Several methods are currently used to determine EV concentration in a sample. Nanoparticle Tracking Analysis (NTA) and Tunable Resistive Pulse Sensing (TRSP) are the most commonly used methods to estimate particle size and concentration. However, these methods are unable to differentiate between EV and non-EV particles, therefore, additional techniques that allow to identify EV structural properties and single particle EV visualization should be used in conjunction with these techniques.

All types of EVs can be detected and characterized at the level of a single EV using electron microscopy (EM). Transmission electron microscopy (TEM) and scanning electron microscopy (SEM) are considered a standard tool for characterizing EV morphology. The additional immuno-labeling using nanogold particles allows the detection of one or more EV surface proteins, which can be differentiated by varying the size of the nanogold particle, thus allowing for multiplex labeling. However, to visualize EVs within cells or tissues with EM techniques requires a high level of manipulation of the sample, while isolated EVs need to be dehydrated and stained, affecting the overall structure of the EV. Nevertheless, the emergence and fine-tuning of the cryo-electron microscopy (Cryo-EM) technique has emerged in order to reveal detailed structural features in EVs allowing to determine morphological heterogeneity. Thus, cryo-EM identifies EVs by their lipid bilayer and allows to differentiate them from non-vesicular bodies with little sample manipulation. EVs can be visualized either dried or hydrated or unstained using a thin film of frozen liquid with cryo-EM (Coumans et al., 2017). Interestingly, this technique has allowed to identify a percentage of exosomes derived from human melanocytes presenting a cap structure consisting of a stack of horizontal layers, unveiling novel EV morphology (Van Niel et al., 2015). In fact, the use of cryo-electron tomography has allowed to obtain enhanced imaging of EV as it generates 3D images showing the spherical morphology of exosomes (Peters et al., 2006; Van Niel et al., 2015). An additional technique that minimizes sample preparation is Atomic force microscopy (AFM), which allows to obtain surface topographic 3D images (Whitehead et al., 2015; Figure 1B).

### EV labeling and uptake visualization

EV uptake relies mainly on scoring fluorescent or bioluminescent signals in cells or tissues treated with labeled purified EVs. Different membrane-specific fluorescent dyes are being used for this purpose such as PKH67, PKH26, DiI, DiR, and rhodamine B (Hoshino et al., 2015; Szempruch et al., 2016; Kamerkar et al., 2017; Ying et al., 2017; Figure 1C). Anyhow, the use of these compounds has some limitations as: (i) they can affect normal EV behavior, (ii) they label all EV aside from their origin making it difficult to discern a specific EV population, (iii) they are not suitable for long term studies due to their short half-life, and (iv) they can label not only EVs but can also stain aggregates and/or form micelles giving false positive results (Lai et al., 2015). The uptake of EVs can also be measured using fluorescence microscopy, flow cytometry or more advance single-cell cytometry techniques such as the ImageStream® Flow Cytometer (Clark, 2015). It is important to consider, that some cells may not internalize EVs and signal through EV-plasma membrane receptor cascades. To confirm that EVs are not simply attached to the cell surface and that they are binding the plasma membrane, trypsin or acid treatments of the recipient cells must be applied, although these methods will influence EV-cell functionality (Franzen et al., 2014).

In order to overcome the limitations of the short half-life that lipid-membrane fluorescent dyes have, Lai et al. developed a fluorescent EV labeling strategy to achieve live-cell imaging of EV release, uptake, and exchange between different cell populations, as well as microscopic quantification and flow cytometry analysis (Lai et al., 2015). For the generation of fluorescent EV reporters, a palmitoylation signal is genetically fused in-frame to the N-terminus of enhanced green fluorescence protein (EGFP) and tandem dimer Tomato (tdTomato), generating PalmGFP, and PalmtdTomato labeled EVs. Cells are then transduced with a vector encoding either PalmGFP or PalmtdTomato. This approach allows to: (i) label multiple EV types irrespective of their biogenesis, (ii) evaluate time-lapse live-cell imaging of EV release and uptake, and (iii) determine EV exchange between different populations (Lai et al., 2015). Furthermore, the authors also fluorescently labeled mRNA and quantified mRNA EV transfer between cells (Lai et al., 2015). Another clever strategy this group used is to fuse luciferase to a transmembrane protein, allowing to determine EV transfer by measurement of the luciferase activity in the recipient cells (Lai et al., 2014). Alternatively, tetraspanins such as CD63, have been fused to fluorescent proteins originating fluorescently labeled EVs (Lo Cicero et al., 2015; Sung et al., 2015). The EV concentration can then be determined by fluorescence correlation spectroscopy to enable quantification at the single vesicle level (Heusermann et al., 2016), confocal, or stimulated emission depletion (STED) super-resolution microscopy to determine the rapid binding and incorporation of EVs in the target cell (Cossetti et al., 2014). Besides, and in the same direction as this, bioluminescent reports have been used to label EVs for *in vivo* studies. As an example, Baglio et al. developed a bioluminescent orthotopic xenographt mouse model to investigate whether osteosarcoma EVs alter the physiology of mesenchymal stem cells (MSCs) such that they promote tumor progression. Briefly, luciferase-positive metastatic osteosarcoma cells were inoculated in a tibia of immunocompromised mice; human GFP-positive MSCs were educated with osteosarcoma-released EVs for 48 h, and educated or non-educated MSCs were systematically injected in the osteosarcoma-bearing mice. Tumor growth was later monitored by bioluminescence imaging (BLI) (Baglio et al., 2017).

However, there is a need to develop methods that allow discriminating between the cells that have taken up EVs and the ones that have not, in the same microenvironment. The Cre-*lox*P method was designed to specifically identify cells taking up EVs (Figure 1C). The Cre-*lox*P system induces a color switch in reporter-expressing cells that take up EVs released from cells expressing the recombinase Cre; i.e., the recipient cells that do not take up exosomes express a DsRed reporter, while the cells that take up EVs start expressing GFP (recombined reporter as they have taken up Cre expressing EVs). Importantly, this system has been proven to efficiently determine EV uptake not only *in vitro* but also *in vivo* (Zomer et al., 2015, 2016). Furthermore, mRNA transfer of Cre recombinase has also been similarly shown by a different group in the context of inflammation (Ridder et al., 2014, 2015).

### EV cargo identification

Different studies have focused on providing a comprehensive characterization of the content of EVs. It has been published that EV cargo includes nucleic acids, lipids, proteins and, more recently, metabolites from donor cells (Figure 1D). Different techniques have been used in the last years in order to identify their content and several public datasets have been created to share with the scientific community (Kalra et al., 2012; Kim et al., 2015).

Numerous groups have analyzed the presence of nucleic acids in EVs (Valadi et al., 2007). The amount of RNA and DNA varies depending on the cell of origin although some studies have found little correlation between cellular and EV RNA content (Nabet et al., 2017). Microarray assessment and next-generation sequencing techniques (Eirin et al., 2014) have shown that EVs contain messenger RNA, in addition to both short and long RNAs. Interestingly, a recent study has found an enrichment of non-coding RNAs in exosomes including miRNAs compared to cellular RNA (Nabet et al., 2017). Many other RNAs are also present in EV such as short ncRNAs (miRNAs, piRNAs, and tiRNAs), mid-size ncRNAs (snoRNAs, PASRs, TSSs-RNAs, and PROMPTs), and long ncRNAs (lincRNA, T-UCRs, and others; Pegtel et al., 2010; Nolte-'t Hoen et al., 2012; Quek et al., 2015; Tosar et al., 2015; Sharma et al., 2016; Lee et al., 2017). Although, several studies have shown the presence of different types of RNAs, the international society for extracellular vesicles (ISEV) have reported a list of experimental details that should be present in publications regarding the composition and function of RNA associated to EVs. Particular emphasis is made regarding the possibility that they are contaminants and not within the EV, the challenges of dealing with low amounts of material and further *in vivo* functional validation and characterization (Mateescu et al., 2017). In addition to RNA, also genomic DNA has been detected inside EVs (Balaj et al., 2011; Thakur et al., 2014). A comparison made by Thakur et al. showed that DNA extracted from intact EVs and EVs pre-treated with DNase decrease in double-stranded DNA longer than 2.5 kB in the fraction subject to enzymatic cleave. Therefore, EV isolation for DNA analysis should, nowadays, include external DNase digestion (Miranda et al., 2010). Furthermore, EVs can contain pseudogenes and transposable elements such as retrotransposons, although their biological relevance is still unknown (Balaj et al., 2011; Lefebvre et al., 2016). The commonly used techniques to determine nucleic acid cargo within EVs are next-generation sequencing. However, several steps of the process such as the RNA extraction, library preparation, cDNA synthesis, adapter ligation and different sequencing platforms used can bias the end point result (Goodwin et al., 2016; Mateescu et al., 2017). Ideally, more sensitive techniques that allow the detection of low abundance of certain nucleic acids in EVs or the identification of nucleic acid content in single EVs would prove extremely useful to improve our knowledge of the relevance and functionality of nucleic acids as EV cargo.

Protein and lipid cargo of EVs have been studied via biochemical assay and mass spectrometry (MS) (Haraszti et al., 2016). Protein content study can be performed using a number of different antibody-based assays for the detection of specific proteins but, in the last years, techniques based on advanced mass spectrometry are used to reflect the complete proteome (Raimondo et al., 2011; Colombo et al., 2014). The key step for the development of a robust antibody-based assay is the availability of highly specific antibodies that bind the target. In spite of the antibody specificity limitation, these assays provide important information concerning protein composition of the EVs. Here we can include flow cytometry, EV array (Jorgensen et al., 2015), surface plasmon resonance imaging (SPRi) combined with antibody microarray (Zhu et al., 2014) or nanoplasmonic exosome assay (nPLEX) (Im et al., 2014). Standard proteomic approaches are employed to examine EV protein cargo, ranging from two-dimensional gel electrophoresis to more sophisticated MS techniques like electrospray ionization (ESI)-based liquid chromatography and tandem MS methods such as LC-MS/MS (Schey et al., 2015). Furthermore, the development of more sensitive quantitation methods as the use of metabolic labeling like stable-isotope labeling by amino acids in cell culture (SILAC) or isobaric tags for relative and absolute quantitation (iTRAQ) allow more fine-tuned analysis of the EV proteomic content (Guenther et al., 2015).

Although, numerous proteomic studies reveal EV protein content, only a few researchers have focused their work on the lipid profiling of EVs (Del Boccio et al., 2012). Lipidomics is defined by the characterization and quantification of lipid species in biological samples (Kreimer et al., 2015). The methods currently available to provide information about lipid composition in EVs are high-sensitivity mass-spectrometry-based approaches including liquid chromatography and gas chromatography coupled to MS (Subra et al., 2010).

Recently, a new-omics approach has been linked to the field. Metabolomics strategy provides the characterization of EVs intrinsic metabolic activity applying state-of-the-art untargeted and targeted metabolomics tracing analysis (Iraci et al., 2017; Figure 1D). Indeed, Iraci et al. have found that EVs derived from neural stem cells (NSC) are able to consume and produce metabolites. In particular, this elegant study shows that EVs contain L-asparaginase activity and function as independent metabolic units to alter the microenvironment (Iraci et al., 2017).

## Conclusions

Although, our advance in the understanding of EV biology has impressively evolved in the last decades, we are still far from acquiring a comprehensive knowledge of the basic mechanisms regulating EV biology in both physiological and pathophysiological contexts. Despite new technological advances to study EV biology evolving regularly, further high sensitive techniques will be required to complete our knowledge of EV biology. For the moment, the use of a variety and combination of different standard and novel techniques will help improve our understanding of EVs structure, cargo, function, and biological relevance in different contexts.

## Author contributions

PC-F and JF-L wrote and edited the manuscript. AO suggested the topic and edited the manuscript with input from all the authors.

### Conflict of interest statement

The authors declare that the research was conducted in the absence of any commercial or financial relationships that could be construed as a potential conflict of interest.
